# The complete mitogenome of ‘Pingbancai’, an important economic *Saccharina* cultivation variety

**DOI:** 10.1080/23802359.2016.1186517

**Published:** 2016-07-10

**Authors:** Jing Zhang, Benjie Gao, Tao Liu, Lei Zhang, Na Liu

**Affiliations:** a College of Biology Engineering, Qilu University of Technology, Jinan, Shandong, China;; b College of Marine Life Sciences, Ocean University of China, Qingdao, Shandong, China

**Keywords:** Conservative evolution, mitogenome, phylogenetic relationship, Pingbancai

## Abstract

*Saccharina* is one of the most important economic seaweed. We presented the complete mitogenome of *Saccharina* variety ‘Pingbancai’ (*Saccharina japonica* ×* latissima*) in this work. Circular-mapping mitogenome was 37,658 bp in length with overall A + T content of 64.70%, encoding 3 rRNAs (23S, 16S and 5S), 25 tRNAs, 38 genes (including 3 open reading frames, ORFs). Gene arrangement and component of ‘Pingbancai’ mitogenome were identical to those *Saccharina* species and cultivation varieties, which show highly conservative evolution in mitochondrial genomes within *Saccharina*. The phylogenetic analysis based on mitogenomes showed that ‘Pingbancai’ had a closer evolutionary relationship with *Saccharina japonica* than *Saccharina latissima* and the supported mitogenome was maternally inherited.

*Saccharina* (Laminariales, Phaeophyceae) have become an important raw material of the marine chemical industry (Jensen 1993). In China, more than 10 breeding varieties have been bred (Li et al. 2007; Zhang et al. 2011b), ‘Pingbancai’ is one of the most important economic *Saccharina* cultivation varieties. Here, we determine the complete mitogenome of ‘Pingbancai’ and construct phylogenetic tree of Laminariaceae, providing new molecular data for population diversity and phylogenetic study.

One specimen of ‘Pingbancai’ (accession number: 201004472) was collected from Li dao Bay, Shandong, China and stored at −80 °C in the Culture Collection of Seaweed at Ocean University of China. The experimental protocol and data analysis methods followed Zhang et al. (2011a).

The complete mitogenome of ‘Pingbancai’ was characterized as a circular molecule of 37,658bp (GenBank accession number KX073817) with a nucleotide composition of 28.42% A (10,701), 14.72% C (5543), 20.58% G (7751) and 36.28% T (13,663). The mitogenome had an overall A + T content of 64.70%. Cumulative GC-skew (0.1660) and AT-skew (–0.1216) analysis of mitogenome reflected a slight bias towards G and T in nucleotide composition on H-strand. The mitogenome encoded 66 genes, including 3 rRNAs, 25 tRNAs, 35protein-coding genes and 3 ORFs. The gene arrangement and component were identical within *Saccharina* mitogenomes (Yotsukura et al. 2010; Zhang et al. 2011a, 2013), showing a highly conservative evolution. Excepting *rpl*2, *rpl*16, *rps*3, *rps*19, *tat*C and ORF130, 60 genes were encoded on H-strand. All protein-coding genes used standard codon ATG as their initial codon. All three typical stop codons are used with a preference of 68.42% for TAA, compared with 21.05% for TAG and 10.53% for TGA. This mitogenome carries 2445bp of intergenic regions accounting for 6.49% of the genome and 13 pairs of overlapping genes with the overlap size from 1 to 16bp were conserved in *Saccharina* mitogenomes. Moreover, all tRNA sequences were potential to form standard cloverleaf secondary structures.

All Laminariaceae species with complete mitogenomes available in the GenBank were selected to construct the phylogenetic tree by the Bayesian method. Phylogenetic analysis based on combined 35 protein-encoding genes exhibited the species were divided into two clades: *Saccharina* and *Laminaria* (Figure 1), supporting current taxonomic systems (Yoon et al. 2001; Lane et al. 2006). It also showed that ‘Pingbancai’ firstly groups with *S. japonica* and then with *S. latissima* and validated that the mitogenome was maternally inherited.

**Figure 1.**
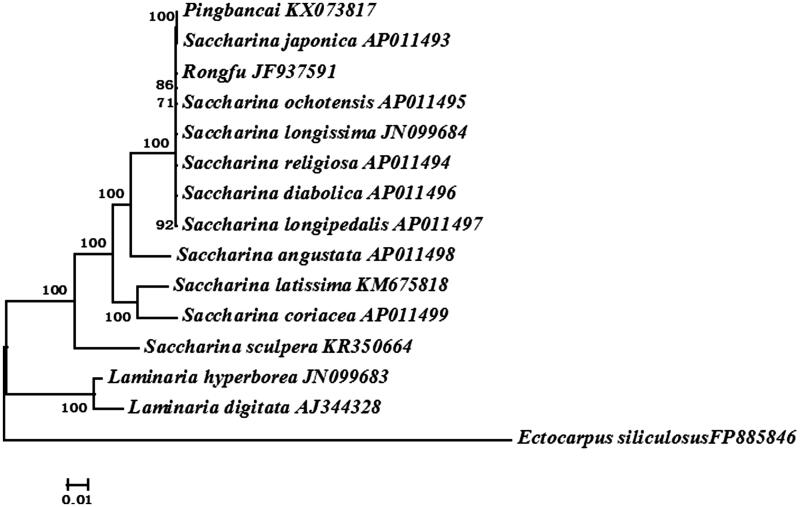
Phylogenetic trees derived from the Bayesian analysis constructed based on concatenated nucleotide sequences of 35 mtDNA protein-encoding genes.
